# Food Fortification: The Level of Awareness among Kenyan Consumers

**DOI:** 10.1155/2020/8486129

**Published:** 2020-04-07

**Authors:** Amaya Aura Linda, Florence Kyallo, Judith K. Okoth, Peter Kahenya, Anselimo Makokha, Daniel Sila, John Mwai

**Affiliations:** ^1^Department of Human Nutrition Sciences, Jomo Kenyatta University of Agriculture and Technology, Juja, Kenya; ^2^Nutrition and Dietetics Unit, Ministry of Health, Nairobi, Kenya

## Abstract

More than half of the morbidity and mortality cases among children in Kenya are as a result of micronutrient deficiencies (MNDs). Food fortification is considered by the Government of Kenya as a feasible strategy for addressing MNDs. Worldwide, fortification has been proven to be effective since it does not require any change in dietary habits. Success of large-scale food fortification however may depend on consumer awareness of the fortification benefits. A cross-sectional study was conducted in 13 counties to collect information on fortification awareness using structured questionnaires. 1435 respondents were selected using the Lot Quality Assurance Sampling method. Data were analyzed using Stata version 14.0 and statistical significance *p* < 0.05. The study participants were described using descriptive statistics. The association of sociodemographic characteristics and awareness of fortification was performed using binary logistic regression analysis. The median age of the study participants was 35 years. Only 28% of the respondents were aware of the term “fortification.” Of the respondents, about 27% heard of food fortification through radio. Vernacular radio emerged as the most preferred channel for communicating fortification information among 24.9% of the respondents. Although awareness of vitamins (32%) and minerals (1.5%) was limited, most (76%) respondents reported of existence of health risks for lacking micronutrients. Awareness of food fortification was significantly associated with respondents' occupation (*p* <  0.001), household size (*p*=0.012), education levels (*p* < 0.001), and age (*p*=0.025). There is need for a wider use of broadcast media sources to modify information and education materials to promote fortification awareness among Kenyan consumers.

## 1. Introduction

Micronutrient deficiency, or “hidden hunger,” is regarded a significant contributor to the global burden of disease. It is estimated that over 2 billion people in the world today are micronutrient deficient particularly vitamin A, iron, iodine, folate, and zinc [1]. In Kenya, like other developing countries, malnutrition continues to raise morbidity and mortality concerns. More than half of the morbidity and mortality cases especially among children are as a result of zinc, iron, and vitamin A deficiencies [2]. The main forms of micronutrient deficiencies in Kenya include vitamin A, iron, folate, vitamin B12, iodine, and zinc deficiencies [2]. According to the KNMS 2011, 83.3% of preschool children are zinc deficient. Iron deficiency is at 36.1% in pregnant women and 21.8% in under 5 children. The national prevalence of vitamin A deficiency (VAD) is 4.1% with the margin at risk for under 5 children being at 52.6%. National folate deficiency is at 32.1% in pregnant women and 30.9% in nonpregnant women, while 22.1% of school-age children are iodine deficient.

In recognition that micronutrient deficiency remains an obstacle to the overall national development, the Government of Kenya (GoK) adopted and mandated fortification of certain staple foods in 2012 including salt, wheat flour, maize flour, and vegetable oils and fats to specific legal standards [3]. This approach has been shown to increase access to micronutrients of public health significance without the need for drastic changes in consumption patterns [4]. Consumer perception of fortified foods and consumption of these fortified products, however, may depend on their knowledge and awareness on nutritional issues [5]. Thus, this makes it important to understand barriers to demand and consumption of fortified foods that relate to consumer awareness about the importance of micronutrients [6]. Studies that have focused on consumer levels of awareness on food fortification in developing countries are scanty. Yet, an increasing number of developing countries such as Kenya, Uganda, Tanzania, Malawi, and Zambia are promoting nutrition policies that integrate food fortification as part of the strategies for preventing micronutrient deficiencies [7]. Therefore, this study aimed to assess awareness levels of Kenyan consumers on food fortification.

## 2. Materials and Methods

### 2.1. Study Design and Selection of Study Participants

A cross-sectional study was carried out to collect consumer data from 1,435 participants in 13 counties of Kenya (Table 1). Multistage cluster sampling method was used to group the population into 9 regions. Cluster sampling was used in selecting 13 counties from the 9 regions. Within the counties, systematic sampling was done in selecting 67 subcounties, 67 wards, 67 locations, and 67 sublocations. From the 67 sublocations, a total of 76 villages were randomly selected to achieve the desired sample size. Finally, the simple random sampling method was used in selecting households for interviews. The sample size was determined using the Large Country-Lot Quality Assurance Sampling (LC-LQAS) method, whereby the standard sample size is 19 respondents per enumerated area [8]. The inclusion criteria for the study participants were as follows:

  Household head or a household member who is above 15 years of age
  Permanent residents-respondents that have stayed in the enumerated area for more than three months
  Respondents that accepted to give consent to participate in the study

The exclusion criteria for the study participants were as follows:

  A household member who is below 15 years of age
  Temporary residents
  All the selected subjects who did not consent to participate in the study

### 2.2. Data Collection

Structured questionnaires which had been pretested were administered to the study participants. The questionnaires were used to collect primary data on household sociodemographic characteristics and awareness of food fortification.

### 2.3. Ethical Approval

Ethical clearance was obtained from the Ministry of Health (MoH). Permission to conduct household interviews was obtained from the county officials in the respective counties. Ward administrators and village elders who served as guides during household interviews were contacted in the respective enumerated areas as well. Finally, the primary survey respondent also gave consent to be interviewed on the basis that participation was voluntary.

### 2.4. Survey Tool

The survey tool used for data collection was the structured pretested questionnaire. Mobile data collection platform was used to ensure quality data are collected. Structured questions on the ODK system in the tablets were ordered in a logical sequence and were both closed and open-ended.

The questionnaire gathered information on sociodemographic characteristics, food fortification awareness, source of food fortification information, preferred channel for communicating food fortification information, and finally awareness of micronutrients and health risks for lacking these micronutrients.

### 2.5. Data Processing and Analysis

Unstructured questions that were not coded were coded into the computer as variables after which they were entered, cleaned, and analyzed using STATA version 14.0. Descriptive statistics was used to provide the general characteristics of the study participants. The association of sociodemographic characteristics and awareness of food fortification was done using binary logistic regression. Statistical significance was set at *p* < 0.05.

## 3. Results

### 3.1. Sociodemographic Characteristics of the Households

A total of 1,435 participants were interviewed. More females were interviewed (76%) than males (24%). The median age of the respondents was 35 years (Table 2). A considerably high proportion (65.2%) of the respondents were aged between 25 and 49 years. About half of the respondents (49.3%) had attained primary education. The majority of the respondents (73%) were married. Regarding the household composition, 34.7% of the respondents had about 3-4 dependents. Almost half (40%) of the respondents were reported to be self-employed.

More than three-quarters (76%) of the households were male-headed. About 44.7% of the household heads had achieved primary education. Less than half of the house heads (about 41%) were self-employed. More than half (59%) of grocery shopping was done by wives.

### 3.2. Awareness on Food Fortification

Less than one-third (28%) of the respondents were aware of the term “food fortification.” Female respondents (COR = 0.7; CI, 0.6–1.0; *p* value = 0.023) were likely to be more aware of food fortification than the male respondents (Table 3). Respondents aged 18–24 years (AOR = 0.3; CI, 0.1–0.9; *p* value = 0.025) and greater than 50 years (AOR = 0.3; CI, 0.1–0.9; *p* value = 0.042) were more likely to have food fortification awareness compared to respondents below the age of 18 years. Secondary and tertiary education increased the odds of being aware of the term food fortification. Respondents who had secondary education were 2.3 times more likely to be aware of food fortification compared to respondents without any formal education. Respondents who had tertiary education were 3.2 times more likely to be aware of the term food fortification compared to respondents without any formal education. Households with more than seven dependents had significant levels (*p*=0.012) of food fortification awareness compared to households with one or two dependents. Respondents that were in formal employment were 2 times more likely to be aware of food fortification compared to their counterparts who reported to be house husbands/wives.

### 3.3. Sources of Information on Food Fortification

**Figure 1:**
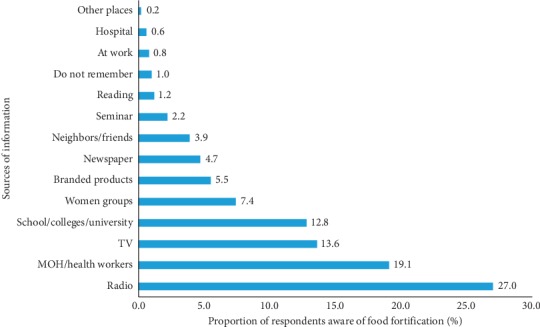
Sources of food fortification information.

Respondents who had heard or read something about food fortification were also asked about the source of that information. Barely, a third (27%) of these respondents reported having received food fortification information through radio (Figure 1). This was followed by the Ministry of Health and television channels reported by 19% and 13.6% of the respondents, respectively. Other sources of food fortification information identified by the respondents included tertiary institutions, women groups, food labels on packaging material, newspapers, friends, and conferences/seminars.

### 3.4. Preferred Channels for Communicating Food Fortification

**Figure 2:**
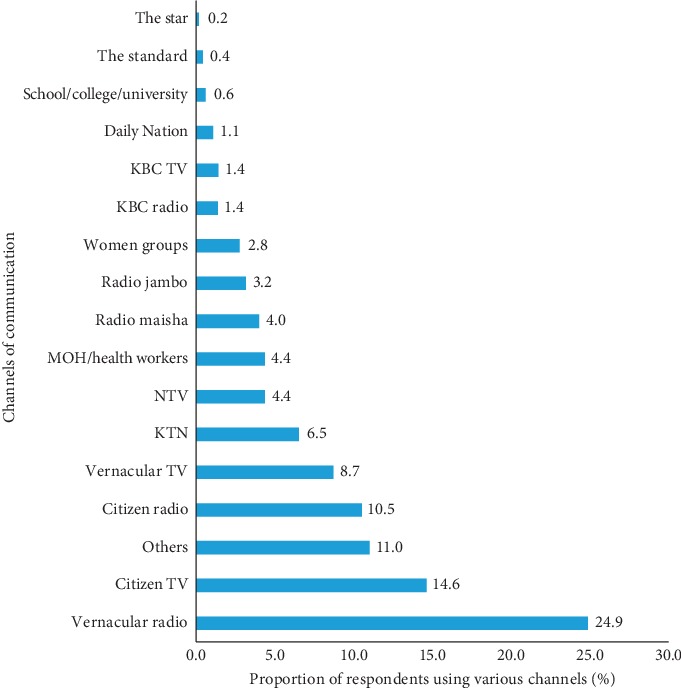
Respondents' preferred channels for communicating food fortification.

Vernacular radio emerged as the forefront preferred channel for communicating food fortification information among 24.9% of the respondents (Figure 2). This was followed by citizen television (14.6%). The others (11%) preferred sources mentioned by respondents including friends, churches, short message services, agricultural extension officers, conferences, and seminars.

### 3.5. Awareness of Micronutrients and Health Risks Associated with Lacking of These Micronutrients

**Figure 3:**
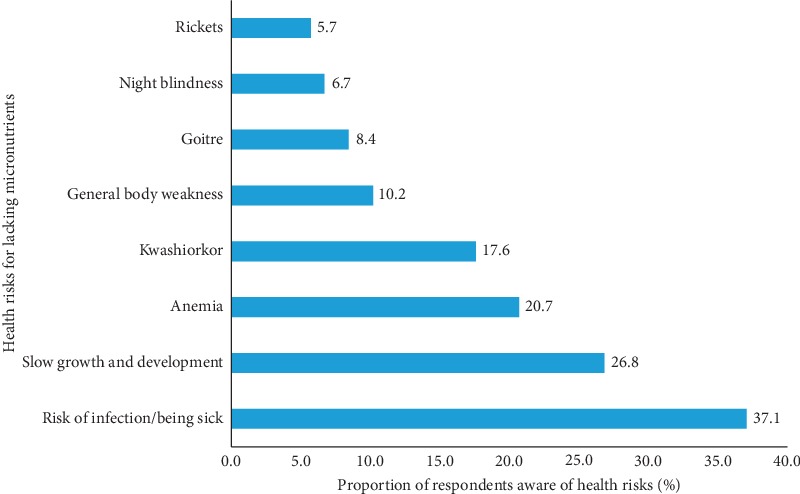
Respondents' awareness of health risks for lacking micronutrients.

Less than half (37.1%) of the respondents were neither aware of vitamins nor minerals. Almost a third (32%) and about 1.5% of the respondents were aware of vitamins and minerals, respectively. Awareness of both vitamins and minerals was low. Respondents who were aware of vitamins mentioned vitamins A, B, C, D, E, and K and further stated milk, vegetables, such as spinach, and fruits to be the best sources of these vitamins. Also, respondents who were aware of minerals stated calcium, iodine, iron, phosphorus, zinc, and potassium. Some went further and revealed the sources for these minerals which included salt, fish, fruits, and vegetables such as cabbages. Furthermore, most (76%) respondents acknowledged that there are health risks associated with consuming insufficient amounts of vitamins and minerals. Risk of infection as a health risk for lacking micronutrients was indicated by 37.1% of the respondents (Figure 3). This was followed by slow growth and development and anemia among 26.8 and 20.7 percent of the respondents, respectively.

## 4. Discussion

In this study, the majority (72%) of our respondents had heard of food fortification for the first time during interviews. This is consistent with a study done in Australia that reported recognition of the term “fortification” to be very low among consumers although the concept of adding vitamins and minerals to foods was generally known [9]. Females were more likely to be aware of food fortification initiatives compared to males. The authors of gender differences in food choice and dietary intake in modern societies argue that women engage in health-promoting information and have healthier lifestyle patterns to a greater degree than men who often show skepticism and resistance to nutrition education messages [10]. Thus, program implementers should promote gender roles when conducting nutrition education programs. Older consumers have been shown to be more aware of nutrition information due to their accumulated experience in food purchase activities [11]. Contrary to the expectations, younger people may also be exposed to numerous modern technology-based channels of information dissemination including phones and media. Therefore, respondents younger than 18 years were also included in this study. Formal education of consumers has been demonstrated to be effective in dissemination of nutrition education since it enhances grasp [11]. The results of this study show significant levels of food fortification awareness among respondents that attained secondary (*p*=0.002) and tertiary education (*p* < 0.0001). Thus, the attainment of higher education can increase the ability to understand and store nutrition information long enough as memory and later use it to a food-related decision [12].

Presence of dependents especially young children in the household might positively influence consumer awareness of food fortification. This is because parenting triggers focus on nutrition and quality consciousness of foods [9]. Therefore, shoppers with children are likely to be more aware of food fortification initiatives which influence them to look for healthy foods when making purchase decisions. Formal employment is an indicator of being well educated reflecting a high socioeconomic status that may influence preference for nutritious foods [13]. These results share a number of similarities with Verbeke findings that reported besides age, gender, and presence of young children, higher education attainment reinforces the idea of a cognitive-oriented decision-making process including active reasoning for functional foods [13]. In addition, some places of work provide their staff with nutrition information. Thus, consumers in formal employment are likely to be more aware of nutritional issues compared to their counterparts who are unemployed.

Regarding the sources of food fortification information, our results confirm Groote and Kimenju's findings that reported radio as one of the most important sources of information, with 79% of the respondents in that study having reported of listening to radio daily [14]. The same study further explained that the preferred language varies with education and ethnic background of the consumers. People with secondary or tertiary education tend to listen to English language programs, whereas people with primary education listen more to Kiswahili or vernacular language programs [14]. It is very likely that the radio can be an effective channel for communicating food fortification information. In this study, the preference for vernacular radio stations could be attributed by low levels of education reported by the respondents. It is important for program planners to take advantage of this channel to create awareness especially among the poorer households for them to appreciate the fact that some of the food products they purchase are fortified.

Awareness of micronutrients was limited among our study respondents. Our results have a number of similarities with Rowland et al.'s findings that reported low awareness of vitamins and minerals among Australian consumers, having identified vitamins C, B-group, and D and minerals (calcium and iron) [9]. Despite the low levels of education, majority (76%) of our respondents acknowledged the existence of health risks associated with consuming inadequate amounts of micronutrients. FSANZ report showed that Australian and New Zealanders women of childbearing age irrespective of their education status were more likely to be aware of health risks associated with lacking essential micronutrients [15]. Considering that females comprised of the high proportion (76%) of our respondents, this could have similarly attributed to our findings.

## 5. Conclusion

In general, there was limited awareness of food fortification among the study participants. The findings of this study suggest that formal education supports awareness concerning nutritional issues. Increasing consumer awareness levels may improve food fortification communication through schools. This calls for collaboration among health and educational officials, teachers, students, parents, and community leaders in fostering health through improvement of learning environment, policies, and practices. The study also established that age, household composition, and occupation status significantly influence consumer awareness of food fortification. In addition, marital status had insignificant influence on the levels of consumer awareness. Regarding food fortification communication, much can be achieved through usage of broadcast media sources, health workers, and tertiary institutions in nutrition education. Modification of educational materials should therefore be carefully and specially designed to create awareness.

## Figures and Tables

**Table 1:** Respondents' county of residence selected in the study.

County of residence	Number of households (*n* = 1435)	Percentage
Kakamega	84	5.9
Kilifi	106	7.4
Kisumu	120	8.4
Mombasa	85	5.9
Nakuru	78	5.4
Narok	57	4.0
Trans-Nzoia	131	9.1
Uasin-Gishu	40	2.7
Nairobi	157	10.9
Nyandarua	120	8.4
Meru	54	10.7
Kitui	142	9.9
Garissa	161	11.2

**Table 2:** Sociodemographic characteristics of the households.

Characteristics	Overall *n* =1435 *n* (%)	Female, *n* (%)	Male, *n* (%)
*Age* (yrs)			
15–17	18 (1.3)	14 (1.0)	4 (0.3)
18–24	207 (14.4)	164 (11.4)	43 (3.0)
25–34	472 (32.9)	383 (26.7)	89 (6.2)
35–49	464 (32.3)	352 (24.5)	112 (7.8)
>50	274 (19.1)	184 (12.8)	90 (6.3)
*Level of education*			
No formal education	145 (10.1)	100 (7.0)	45 (3.1)
Primary	707 (49.3)	539 (37.6)	168 (11.7)
Secondary	436 (30.4)	349 (24.3)	87 (6.1)
Tertiary	147 (10.2)	109 (7.6)	38 (2.6)
*Marital status*			
Married	1057 (73.7)	968 (67.5)	89 (6.2)
Divorced/separated	67 (4.7)	14 (1.0)	53 (3.7)
Widowed	116 (8.1)	16 (1.1)	100 (7.0)
Single	195 (13.6)	99 (6.9)	96 (6.7)
*Size of household*			
1-2 dependents	150 (10.5)	—	—
3-4 dependents	498 (34.7)	—	—
5-6 dependents	409 (28.5)	—	—
>7 dependents	378 (26.3)	—	—
*Occupation of the respondent*			
Self-employed	574 (40.0)	420 (29.3)	154 (10.7)
Formal employment	124 (8.6)	93 (6.5)	31 (2.1)
Casual labor	208 (14.5)	139 (9.7)	69 (4.8)
Housewife/husband	331 (23.1)	289 (20.1)	42 (3.0)
Others	198 (13.8)	156 (10.9)	42 (2.9)
House head gender	1435 (100)	344 (24.0)	1091 (76.0)
*House head education level*			
No formal education	147 (10.2)	61 (4.2)	86 (6.0)
Primary	642 (44.7)	169 (11.7)	473 (33.0)
Secondary	457 (31.9)	76 (5.3)	381 (26.6)
Tertiary	189 (13.2)	32 (2.3)	157 (10.9)
*Occupation of the house head*			
Self-employed	588 (41.0)	158 (11.0)	430 (30.0)
Formal employed	256 (17.8)	29 (2.0)	227 (15.8)
Casual labour	340 (23.7)	66 (4.6)	274 (19.1)
Housewife/husband	100 (7.0)	42 (3.0)	58 (4.0)
Others	151 (10.5)	43 (3.0)	108 (7.5)

**Table 3:** Association of the respondents' sociodemographic characteristics and awareness of food fortification.

	Awareness of food fortification		COR (CI 95%)	*p* value	AOR (CI 95%)	*p* value
	Respondents aware, *n* (%)	Respondents not aware, *n* (%)				
*Gender*						
Male	107 (32.9)	218 (67.1)	Ref.		Ref.	
Female	294 (26.5)	816 (73.5)	0.7(0.6–1.0)	0.023	0.8 (0.6–1.1)	0.198
*Age category*						
<18	8 (44.4)	10 (55.6)	Ref.		Ref.	
18–24	52 (25.1)	155 (74.9)	0.4 (0.2–1.1)	0.083	0.3 (0.1–0.9)	0.025
25–34	154 (32.6)	318 (67.4)	0.6 (0.2–1.6)	0.300	0.5 (0.2–1.3)	0.146
35–49	128 (27.6)	336 (72.4)	0.5 (0.2–0.9)	0.127	0.4 (0.2–1.2)	0.107
>50	59 (21.5)	215 (78.5)	0.3 (0.1–0.9)	0.031	0.3 (0.1–0.9)	0.042
*Education level*						
No formal education	24 (16.6)	121 (83.5)	Ref.		Ref.	
Primary	159 (22.5)	548 (77.5)	1.5 (0.9–2.3)	0.114	1.3 (0.8–2.1)	0.270
Secondary	152 (34.9)	284 (65.1)	2.7 (1.7–4.4)	<0.0001	2.3 (1.4–3.8)	0.002
Tertiary	66 (44.9)	81 (55.1)	4.1 (2.4–7.1)	<0.0001	3.2 (1.7–5.8)	<0.0001
*Marital status*						
Single	57 (29.2)	138 (70.8)	Ref.		Ref.	
Married	305 (28.9)	752 (71.1)	1.0(0.7–1.4)	0.915	1.2 (0.8–1.9)	0.294
Divorced/separated	12 (17.9)	55 (82.1)	0.5(0.3–1.1)	0.073	0.7 (0.3–1.5)	0.330
Widowed	27 (23.3)	89 (76.7)	0.7(0.4–1.2)	0.254	1.3 (0.7–2.4)	0.443
*Size of HH*						
1-2 dependents	47 (31.3)	103 (68.7)	Ref.		Ref.	
3-4 dependents	145 (29.1)	353 (70.9)	0.9 (0.6–1.3)	0.602	0.9 (0.6–1.4)	0.593
5-6 dependents	130 (31.8)	279 (68.2)	1.0 (0.7–1.5)	0.919	1.0 (0.7–1.6)	0.867
>7 dependents	79 (20.9)	299 (79.1)	0.6 (0.4–0.9)	0.012	0.7 (0.4–1.1)	0.100
*Occupation*						
Housewife/husband	83 (25.1)	248 (74.9)	Ref.		Ref.	
Self-employed	156 (27.2)	418 (72.8)	1.1 (0.8–1.5)	0.490	1.0 (0.7–1.3)	0.827
Formal employment	53 (42.7)	71 (57.3)	2.2 (1.4–3.4)	<0.0001	1.3 (0.8–2.1)	0.312
Casual labor	51 (24.5)	157 (75.5)	1.0 (0.6–1.5)	0.884	1.0 (0.7–1.5)	0.861
Others	58 (29.3)	140 (70.7)	1.2 (0.8–1.8)	0.289	1.2 (0.7–1.8)	0.519

## Data Availability

The quantitative primary data used to support the findings of this study are included within the article.
